# Upregulated microRNA‐126 induces apoptosis of dental pulp stem cell via mediating PTEN‐regulated Akt activation

**DOI:** 10.1002/jcla.23624

**Published:** 2020-11-05

**Authors:** Rucun Ge, Yongtao Lv, Peipei Li, Lin Xu, Xiaoya Feng, Hongshun Qi

**Affiliations:** ^1^ Laboratory of Regenerative Medicine Shandong Provincial Third Hospital Jinan China; ^2^ Department of Neurology Shandong Provincial Third Hospital Jinan China

**Keywords:** Akt, apoptosis, dental pulp stem cell, miR‐126, PTEN

## Abstract

**Introduction:**

Human dental pulp stem cells (DPSCs) have potential applications in regenerative medicine. The molecular mechanisms underlying DPSCs viability and apoptosis are not completely understood. Here, we investigated the role of miR‐126 in DPSCs viability and apoptosis.

**Material and methods:**

Senescent DPSCs were compared with early passage DPSCs. real‐time PCR and microARRAY were performed to identify the differential expression of miR‐126, and western blot was performed to detect the expression of PTEN. MTT assay was utilized to reveal the proliferative rate of both senescent and early passage DPSCs. Flow cytometry was used to examine the apoptotic rate of DPSCs. Dual‐luciferase reporter assay was carried out to detect the interaction of miR‐126 and PTEN.

**Results:**

Senescent DPSCs showed a high level of apoptosis. Further study showed that miR‐126 is upregulated in senescent DPSCs and its overexpression in early passaged DPSCs induced apoptosis. Phosphatase and tensin homolog gene (PTEN) was identified as a target of miR‐126. PTEN was downregulated in senescent DPSCs, whereas miR‐126 inhibition upregulated PTEN level, and subsequently activated Akt pathway and suppressed the apoptotic phenotype of senescent DPSCs. In addition, PTEN overexpression rescued apoptosis of DPSCs at later stage.

**Conclusion:**

Our results demonstrate that the miR‐126‐PTEN‐Akt axis plays a key role in the regulation of DPSCs apoptosis and provide a candidate target to improve the functional and therapeutic potential of DPSCs.

## Graphical abstract

To probe the role of miR‐126 on cell proliferation and apoptosis of early and later DPSC, the cells at PD16 were transfected with miR‐126 mimic or control, while cells at PD54 were transfected with miR‐126 inhibitor or control to regulate the miR‐126 expression. For early DPSC group, data displayed that there was no any influence, while miR‐126 caused a reduced cell proliferative rate. For DPSC at PD54, miR‐126 inhibition significantly increased the cell replication of DPSC during 72 h post transfection. These data suggested that miR‐126 played an inhibitory role in DPSC viability.

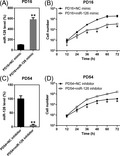

## 1 INTRODUCTION

Oral injuries occupy approximately 17% of all body injuries. Increasing researches have revealed that dental injuries account for 20%‐30% of these injuries, corresponding to 1%‐3% of all body injuries.^1^ Dental pulp is a unique tissue encased in dentin that provides strong mechanical support and protection from bacterial, chemical, and physical stimuli.^2^ Once dentin is disrupted by traumatic injury or advanced caries, the dentin‐pulp complex will exhibit a unique reparative and regenerative potential.^3^

Dental pulp stem cells (DPSCs) are increasingly being recognized as a viable cell source for regenerative medicine.^4^ DPSCs are multipotent stem cells and can differentiate into various cell types including neurons, cardiomyocytes, chondrocytes, osteoblasts, and liver cells, and they are therefore valuable stem cell sources for regenerative therapies for the treatment of dental and other diseases.^5^, ^6^, ^7^, ^8^, ^9^ However, the application of DPSCs in cell therapy is limited by their replicative and differentiation potentials, which are reduced or lost during culturing. Therefore, elucidating the mechanisms underlying DPSCs cell death and apoptosis is important for their application.

MicroRNAs (miRNAs) are a group of non‐coding RNA molecules, with an approximate length of 22‐25 nucleotides, which can post‐transcriptionally regulate the expression of their target genes, ultimately leading to the degradation of the target mRNA.^10^ An increasing number of reports have shown that miRNAs play an essential role in numerous molecular and biological processes, such as apoptosis, cell proliferation, migration, and necrocytosis.^11^, ^12^ MiR‐126, frequently being downregulated in human tumors, has been demonstrated to possess fundamental roles in various cellular processes, especially in the modulation of proliferation and differentiation.^13^, ^14^, ^15^, ^16^, ^17^ MiR‐126 was also involved in the basal function of adipose tissue stem cell and mesenchymal stem cell.^18^, ^19^, ^20^ However, the mechanism of miR‐126 in regulating the function of human DPSCs remain needs to be further investigated. The present study attempts to probe the role of miR‐126 in the features of DPSCs. It appears that expression of miR‐126 was markedly increased in the senescent DPSCs. In turn, senescent DPSCs treated with miR‐126 inhibitor showed apparent upregulated viability and reduced apoptosis. Our findings indicated that miR‐126 could exert a regulatory function in cell viability of senescent DPSCs.

## 2 MATERIAL AND METHODS

### 2.1 Cells and cell culture methods

Normal human third molars were collected from adults (18‐26 years of age) at Qilu Hospital under approval of the Ethics Committee of Qilu Hospital, School of Medicine, Shandong University. All patients gave their written informed consent to participate. DPSCs were obtained as described previously.^21^ Cells were cultured in alpha‐minimum essential medium (a‐MEM; Hyclone, Logan, UT, USA) supplemented with 10% FBS, 100 U/mL penicillin, and 100 μg/mL streptomycin in a humidified atmosphere of 5% CO_2_. The medium was replaced every 2 days. Cells were subcultured at a ratio of 1:3 until they reached 75%‐85% confluence. Population doubling (PD) analysis was performed as described previously.^22^ Cells were declared senescent and apoptotic when they were senescence‐associated β‐galactosidase (SA‐β‐gal) positive and had decreased Bcl‐2, and increased Bax protein expression.

### 2.2 miRNA array

Total RNA was extracted using the phenol‐chloroform method (TRIzol; Invitrogen; Thermo Fisher Scientific, Inc.). The quality of the RNA was assessed by capillary electrophoresis (Agilent Technologies Inc.). Libraries for small RNA sequencing were prepared using the NEBNext Multiplex Small RNA Library Prep Set for Illumina (New England BioLabs, Inc.) according to the manufacturer's protocol. The libraries were quantified using the Agilent Bioanalyzer 2100 system with DNA high‐sensitivity chips. The raw sequence files were subjected to quality control analysis with the Fast QC quality control tool. To avoid low‐quality data, adaptors were removed by Cutadapt (version 1.2.1), and lower‐quality sequences were trimmed. The clean reads were screened at a length of 21‐22 nt as miRNA and were located to the reference sequence with Bowtie software (version 2; CGE Risk Management Solutions BV). The functions of novel miRNAs were analyzed with miRDeep2 software (version 2.0.0.8). Differential expression sequence was used to calculate differential expression levels and to evaluate the statistical significance of detected alterations between the control and case samples.

### 2.3 Western blot

Cells were lysed in lysis buffer (30 mM Tris‐HCl, 150 mM NaCl, 1% NP‐40, and 0.1% SDS; pH 7.4) supplemented with a protease inhibitor cocktail (Roche). The BCA Protein Quantitation Kit (Genescript) was used to determine protein concentration. Proteins were separated using 10% SDS‐PAGE and blotted electrophoretically onto polyvinylidene difluoride (PVDF; Immobilon) membranes of 0.45 µm pore size. Membranes were blocked with 5% bovine serum albumin (BSA) in phosphate‐buffered saline containing 0.1% tween‐20 (PBST) for 1 hour at room temperature, followed by incubation with primary antibody overnight at 4°C: anti‐p21 antibody (ab227443, Abcam), anti‐p16 antibody (ab151303, Abcam), anti‐bcl‐2 antibody (ab59348, Abcam), anti‐bax antibody (ab53154, Abcam), anti‐PTEN antibody (ab31392, Abcam), anti‐Akt antibody (ab8805, Abcam), anti‐pAkt antibody (ab18206, Abcam), and anti‐GAPDH antibody (ab9485, Abcam). HRP‐linked goat anti‐rabbit IgG secondary antibodies (Amersham) were incubated with the membranes for 1 hour at room temperature in PBST containing 5% BSA, followed by chemiluminescent detection. A C‐DiGit Blot Scanner and Super Signal West Femto Maximum Sensitivity Substrate Kit (provided by Thermo) were used to detect bound antibodies.

### 2.4 RNA extraction and Q‐PCR

Total RNA was extracted from tissues using Trizol reagent according to the manufacturer's instructions (Invitrogen). Messenger RNA (mRNA) levels were determined using the Light‐Cycler 480 Real‐Time PCR system (Roche), with GAPDH as the internal control. Quantitative PCR was carried out in 20 μL reaction volumes containing SYBR Green PCR Master Mix for 10 minutes at 95°C; 95°C for 15 seconds, 60°C for 30 seconds, and 72°C for 30 seconds, a total of 40 cycles. Transcript levels were determined relative to the calibrator (mean of the controls) and normalized to the endogenous reference (2^−ΔΔ^ *^C^* ^t^ method).^23^

Sequences of primers used for Q‐PCR detection were showed as follows: PTEN F: 5′‐AAA ATG TCC GTG CAA AGT GGT‐3′, PTEN R: 5′‐CTC GAT CGT GTC TTT ATC ATC CC‐3′; GAPDH F: 5′‐GGA AGG TGA AGG TCG GAG TCA‐3′, GAPDH R: 5′‐GTC ATT GAT GGC AAC AAT ATC CAC T‐3′.

### 2.5 MiR‐126 mimic/inhibitor preparation

Mimic/Inhibitor of miR‐126 and negative control (NC) was acquired from RiboBio. MiR‐126 mimic/inhibitor and NC mimic/inhibitor were supplemented to 0.9% NaCl to a terminal level of 10 mg/mL for further apply.

### 2.6 MTT Assay

The MTT assay was conducted to evaluate cell proliferation. Briefly, cells were treated with 20 μL of MTT (0.5 mg/mL), and the supernatant was discarded. DMSO (150 μL) was then added to each well, with rotation for 10 minutes, to dissolve the formazan dye. An Infinite M200 microplate reader (Tecan) was then used to measure the absorbance at 490 nm.

### 2.7 Annexin V‐FITC/PI flow cytometry

Cell death was evaluated using an annexin V‐FITC/PI apoptosis detection kit. Briefly, after transfection, cells were resuspended in 20 µL of binding buffer and then incubated for 20 minutes with 5 µL of PI and 10 µL of annexin V‐FITC in the dark room. Cell death was evaluated by flow cytometry (FC).

### 2.8 Dual‐luciferase reporter assay

The 3′‐UTR of the PTEN gene underwent amplification prior to fusion with the GV126 luciferase gene. The binding site of the PTEN gene and miR‐126 was mutated via site‐directed mutagenesis, which served as a control. Thymidine kinase promoter (TaKaRa; pRL‐TK vectors) and plasmids containing Renilla luciferase were applied to adjust for transfection efficiency. HEK293T cells were co‐transfected with miR‐126‐mimic and NC‐mimic with luciferase reporter vectors and the luciferase assay was conducted.

### 2.9 Statistical analysis

Data are expressed as mean ± standard deviation (SD). Two‐tailed Student's *t* test was used for mean comparisons. *P* < .05 demonstrated significant difference.

## 3 RESULTS

### 3.1 MiR‐126 is upregulated in DPSCs

**Figure 1:**
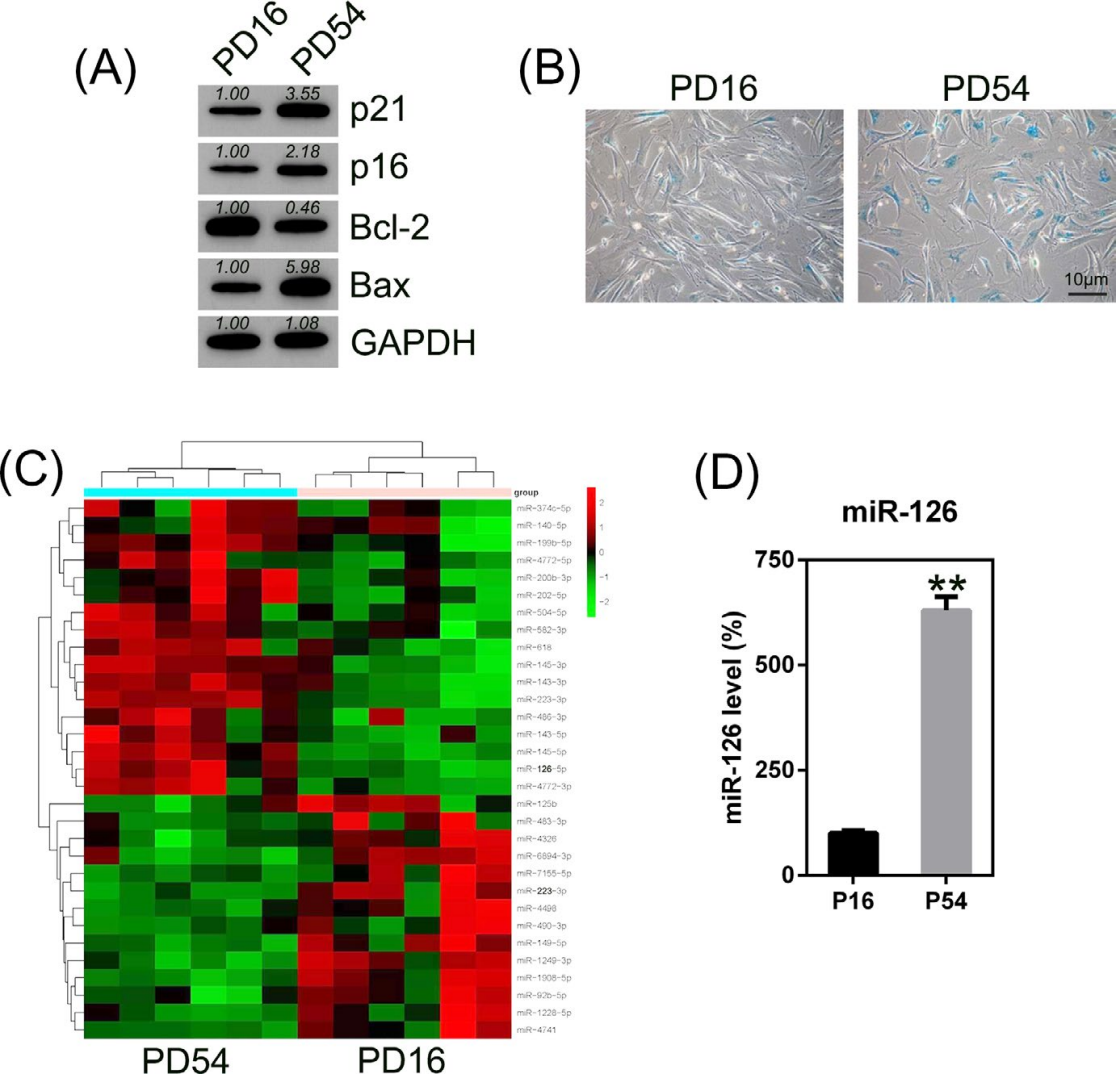
Senescent dental pulp stem cells (DPSCs) show upregulation of miR‐126 expression. (A) WB analysis of p21, p16, Bax, and Bcl‐2 expression in early‐ and late‐passage cells. Bands were quantified by densitometry. (B) Early (PD 16) and later (PD 54)‐passage DPSCs were analyzed by SA‐β‐gal staining. (C) miRNA microarray of dysregulated miRNAs in the early and later DPSCs. (D) miR‐126 expression in early and later cells measured by Q‐PCR. Data are expressed as the mean ± SD from three independent experiments. **P < .01

We extracted DPSCs from normal human third molars which was obtained from adults. Cells were characterized in vitro to confirm their stem characters. Western blot (WB) analysis indicated that the isolated DPSCs were positive for cyclin‐dependent kinase inhibitors p21 and p16, which are markers of senescent DPSCs, were markedly upregulated at PD54 compared with the levels at PD16. We also found that pro‐apoptotic protein Bax was increased while anti‐apoptotic protein Bcl‐2 was decreased in DPSCs at PD54 (Figure 1A), suggesting there existed apoptosis at PD54. The number of SA‐β‐gal‐positive cells increased significantly at PD54 compared with that at PD16, indicating the successful induction of senescent cells (Figure 1B).

The miRNA microarray analysis revealed that miR‐126 was significantly upregulated in PD54 DPSCs, comparing with PD16 DPSCs (Figure 1C). To confirm this data, Q‐PCR analysis was performed for determining the miR‐126 expression at PD16 and PD54. It was found that the level of miR‐126 was upregulated in DPSCs at PD54 (Figure 1D), suggesting that miR‐126 may be played a role in apoptosis of senescent DPSCs.

### 3.2 MiR‐126 is essential for maintaining viability of DPSCs

**Figure 2:**
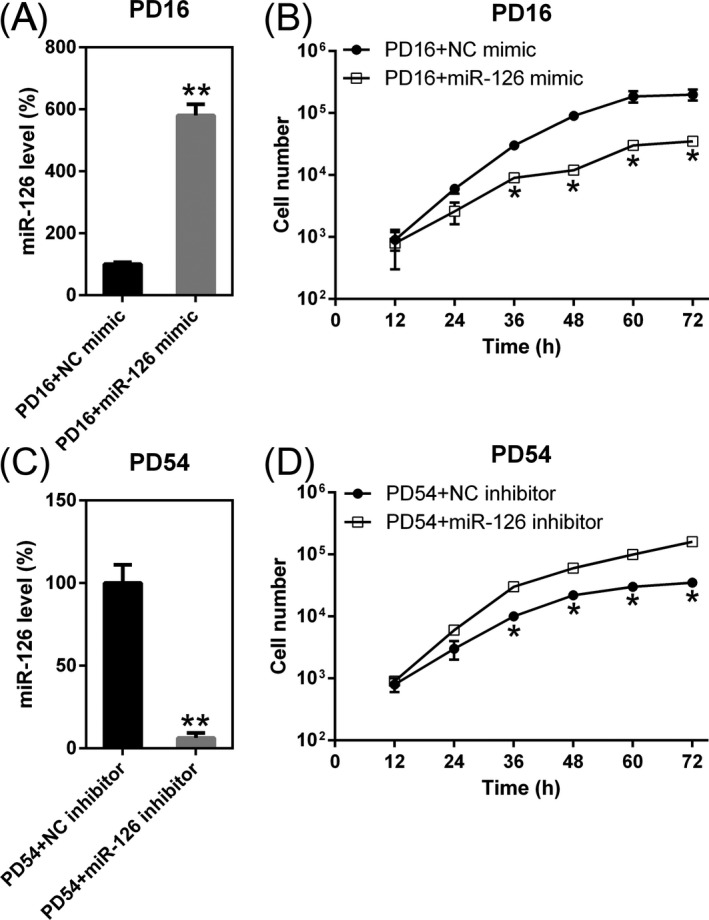
Regulation of miR‐126 expression mediates the cell growth of dental pulp stem cells (DPSCs) at different stages. (A) DPSCs at PD16 were transfected with miR‐126 mimic or its control. At 36 h post‐transfection, Q‐PCR was carried out to detect miR‐126 level. (B) MTT assays were performed to examine proliferation of DPSCs at 12‐72 h subsequent to transfection of miR‐126 mimic. (C) DPSCs at PD54 were transfected with miR‐126 inhibitor or its control. At 36 hrs post‐transfection, Q‐PCR was carried out to detect miR‐126 level. (D) MTT assays were performed to examine proliferation of DPSCs at 12‐72 h subsequent to transfection of miR‐126 inhibitor. Mean ± SD of the results of three independent experiments was used to describe the data. N = 3. **P < .01, compared to indicated group

To probe the role of miR‐126 on cell proliferation and apoptosis of early and senescent DPSCs, the cells at PD16 were transfected with miR‐126 mimic or control, while cells at PD54 were transfected with miR‐126 inhibitor or control to regulate the miR‐126 expression. The Q‐PCR data indicated that usage of miR‐126 mimic increased miR‐126 in DPSCs at PD16 (Figure 2A). Then, we performed MTT assay to detect the proliferative rate of DPSCs during 72 hours post‐transfection. For control group, data displayed that there was no any influence, while miR‐126 caused a reduced cell proliferative rate (Figure 2B). For DPSCs at PD54, miR‐126 inhibition significantly increased the cell replication of DPSCs during 72 hours post‐transfection (Figure 2C,D). These data suggested that miR‐126 played an inhibitory role in DPSCs viability.

### 3.3 MiR‐126 induces apoptosis of DPSCs

**Figure 3:**
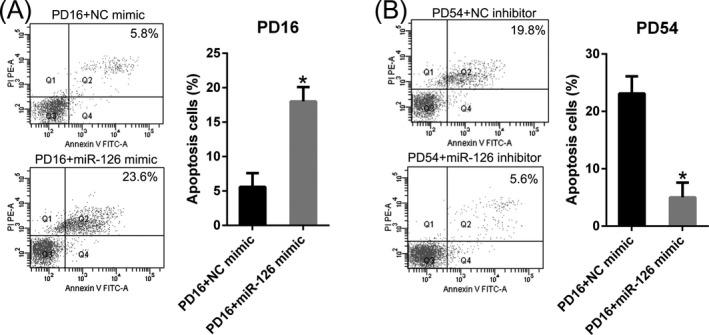
MiR‐126 induced apoptosis of dental pulp stem cells (DPSCs). (A, B) Annexin V‐FITC&PI FC was performed to evaluate the number of apoptotic cells. Early apoptotic cells are showed in the right quadrant of each plot. Analysis of the apoptotic rate of the cells in all groups is displayed in the right panel. Mean ± SD of the results of three independent experiments was used to describe the data. N = 3. *P < .05, compared to the indicated group

We thereby hypothesized that miR‐126 may introduce apoptosis to DPSCs, therefore, caused attenuated DPSCs viability. Therefore, Annexin V‐FITC/PI FC was carried out in cells at different stage following transfection of miR‐126 mimic/inhibitor and NC mimic/inhibitor. DPSCs at PD16 which underwent transfection with the miR‐126 mimic displayed elevated apoptotic numbers of cells at 36 hours post‐transfection, compared with early DPSCs transfected with NC mimic (Figure 3A). Furthermore, Annexin V‐FITC/PI FC also demonstrated that silenced miR‐126 expression remarkably decreased the percentage of apoptotic DPSCs at later stage, comparing with negative control group (Figure 3B). These results suggested that miR‐126 upregulation resulted in a significant increased apoptotic percentage of DPSCs.

### 3.4 MiR‐126 targets PTEN

**Figure 4:**
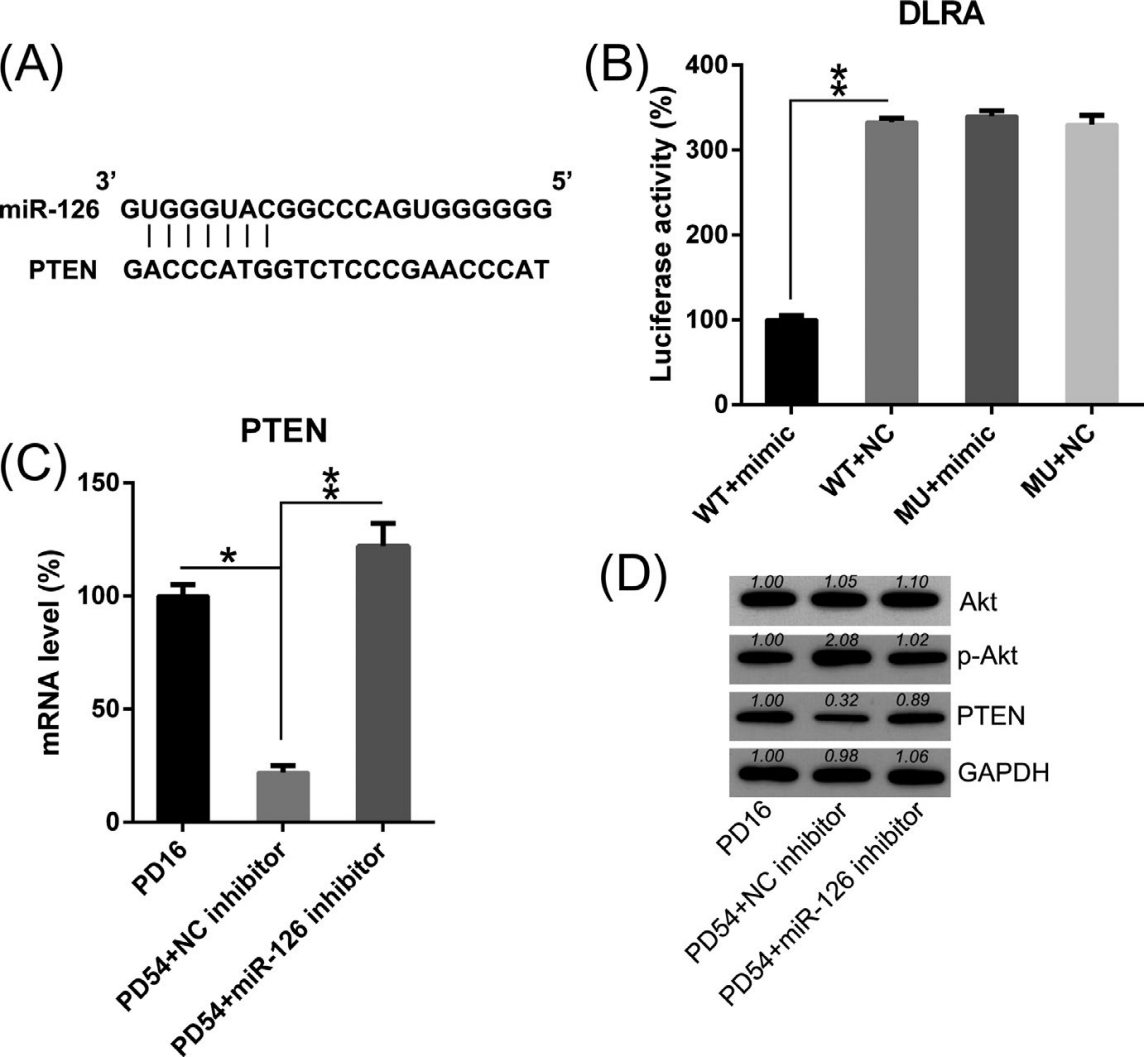
MiR‐126 targets 3′‐UTR of PTEN. (A) Bioinformatics analysis of miR‐126 and 3′‐UTR of PTEN gene. (B) DLRA was performed following co‐transfection with a luciferase reporter containing either a WT (wild‐type) or MU (mutant) 3′‐UTR from PTEN, and a miR‐126‐mimic into HEK293T cells. The effect of miR‐126‐mimic transfection on the luciferase activities of the WT and MU Rac1 reporter constructs was determined. (C) Q‐PCR analysis determined the mRNA levels of PTEN in the dental pulp stem cells (DPSCs) after transfection of miR‐126 inhibitor or NC inhibitor. (D) PTEN, Akt, and phosphorylated Akt levels were determined in DPSCs after transfection of miR‐126 inhibitor or NC inhibitor by WB. Mean ± SD of the results of three independent experiments was used to describe the data. N = 3. *P < .05, **P < .01 compared to the indicated group

It has been reported that PTEN sensor played an important role in modulating apoptotic cell signal.^24^ Bioinformatics analysis predicted that miR‐126 may target 3′‐UTR of PTEN gene (Figure 4A). Transfection of miR‐126 mimic inhibited luciferase function, which was fused with the PTEN 3′‐UTR by 60%, compared to the other control groups (Figure 4B). In light of the key role of PTEN in the apoptosis, we measured its expression in DPSCs by Q‐PCR and WB. Here, the downregulation of PTEN was found in DPSC at PD54, compared with the DPSCs at PD16 (Figure 4C,D). Moreover, PTEN mRNA and protein level were also increased in senescent DPSCs transfected with miR‐126 inhibitor. Furthermore, the downstream Akt phosphorylation was remarkably upregulated in DPSCs at PD54, while transfection with miR‐126 inhibitor caused an impaired Akt phosphorylation in DPSCs. These findings suggest that miR‐126 potentially targets the 3′‐UTR of PTEN, and subsequently influence the signal transduction of PTEN‐Akt axis in DPSCs.

### 3.5 Overexpression of PTEN deprived the apoptotic property in DPSCs

**Figure 5:**
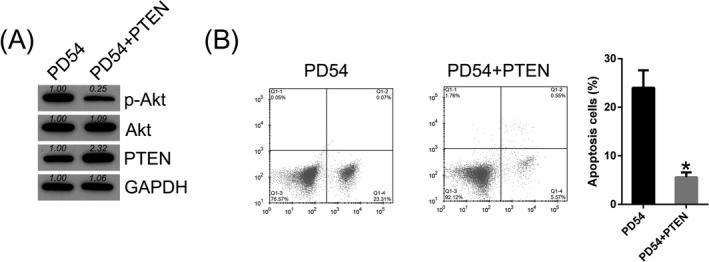
PTEN overexpression reduced apoptosis of senescent dental pulp stem cells (DPSCs). DPSCs at later stage were transfected with PTEN overexpressing vector, or their controls. (A) Protein expression of PTEN, Akt, and phosphorylated Akt levels were determined in DPSCs after transfection using WB. (B) Annexin V‐FITC&PI FC was performed to evaluate the number of apoptotic cells. Early apoptotic cells are showed in the right quadrant of each plot. Analysis of the apoptotic rate of DPSCs in all groups is displayed in the right panel. Mean ± SD of the results of three independent experiments was used to describe the data. N = 3. *P < .05, compared to the indicated group

To determine whether PTEN can play a role in the apoptosis in DPSCs at PD54, PTEN was overexpressed in senescent DPSCs. WB analysis was utilized in order to verify the overexpression in PTEN (Figure 5A). We further showed that overexpression of PTEN significantly deactivated Akt sensor. Annexin V‐FITC&PI FC was performed to evaluate the cell apoptosis in senescent DPSCs with PTEN overexpression. PTEN upregulation in DPSCs at PD54 resulted in a noticeable reduction in apoptotic cells proportion (Figure 5B). These data suggested that PTEN‐Akt signal axis was involved in the apoptosis of senescent DPSCs.

## 4 DISCUSSION

The properties of human stem cells are precisely regulated by intracellular or extracellular mechanical and molecular signals, and are a promising target for stem cell‐based therapies for various human diseases.^25^ Accumulating miRNAs may affect biological reactions via modulation of their target agents. Previous research has demonstrated that miR‐126 may target different genes, including ADAM9,^26^ LRP6, PIK3R2,^27^ CXCR4,^28^ and SLC7A5^29^ which are all involved in the progression of different tumors. However, the regulatory mechanisms of miR‐126 on DPSCs viability remain poorly reported. Therefore, the aim of present study was to explore the effect of miR‐126 on the characteristics of senescent DPSCs, especially on cell viability and apoptosis. Expression of miR‐126 in senescent DPSCs is obviously higher than that in early DPSCs. Meanwhile, senescent DPSCs have an impaired cell viability and enhanced apoptosis. In silico prediction and Dual‐luciferase reporter assay (DLRA), data suggested that miR‐126 targeted 3′‐UTR of PTEN gene, and transfection of miR‐126 inhibitor in senescent DPSCs increased PTEN expression at both protein and mRNA level, as well as attenuated Akt activation. Additionally, PTEN overexpression in senescent DPSCs reduced the apoptotic cell number which was also induced by miR‐126 inhibitor and acts as an essential target for miR‐126 in impairing cell viability.

Previous studies have demonstrated that PTEN‐Akt signaling pathway contributes to the carcinogenesis of variant cancers, such as pancreatic cancer, hepatocellular carcinoma, breast carcinoma, and gastric carcinoma.^30^, ^31^, ^32^, ^33^ Our results suggested that PTEN‐Akt pathway was negatively modulated by miR‐126 in senescent DPSCs, suggesting miR‐126 is a critical negative regulator in transduction of PTEN‐Akt pathway. These findings could be also supported by a previous report^34^ demonstrating the effect of miR‐126 on apoptosis of vascular endothelial cell through targeting PI3K/Akt signaling. To be noted, overexpression of miR‐126 also significantly decreased early DPSCs cell growth, as well as induced cell apoptosis. Using WB and Q‐PCR, we found that the endogenous levels of PTEN in senescent DPSCs were significantly decreased compared with the early DPSCs, in contrast, the activation of Akt in cells was significantly increased, suggesting activation of PTEN‐Akt signal axis may contribute to miR‐126‐attenuated DPSCs viability.

Collectively, our data show that miR‐126 participates in the regulation of cell viability of DPSCs by directly repressing PTEN levels and also indirectly, by activating Akt signal that can function as pro‐apoptotic factors. This study therefore suggests that miR‐126 is a key modulator to maintain the senescent DPSCs phenotype.
